# From mentee to mentor: Becoming an early career professor in psychiatry

**DOI:** 10.1192/j.eurpsy.2021.129

**Published:** 2021-08-13

**Authors:** A. Fiorillo

**Affiliations:** Department Of Psychiatry, University of Campania “L. Vanvitelli”, Naples, Italy

**Keywords:** early career psychiatrist, mentor, Future of psychiatry

## Abstract

In the last years several social, economic and scientific changes have occurred, which have had a significant impact on psychiatric clinical practice, research and training. Some traditional syndromes seem to be disappeared, while new forms of mental health problems are coming to psychiatric consultation. The psychosocial distress caused by the current ongoing crisis due to the COVID-19 or the maladaptive use of the new technologies among the younger generation, are some good examples of psychosocial factors causing new mental health disturbances. Psychiatrists and mental health professionals are not yet well-equipped for managing these, which represent major unmet needs in modern clinical practice. Furthermore, in this evolving social context, the societal role of psychiatrists is completely changed, moving from being “alienist” working in asylums to “mental health care providers” working in the communities. The role of psychiatrists is to improve global mental health and to promote well-being in the general population according to a life-span perspective. Nowadays, early career psychiatrists report several unmet needs that we are not able to manage. In this symposium, I will describe my personal experience of moving from my role of mentee to mentor.

**Disclosure:**

No significant relationships.

